# Resveratrol, a Multitasking Molecule That Improves Skeletal Muscle Health

**DOI:** 10.3390/nu15153413

**Published:** 2023-07-31

**Authors:** Luana Toniolo, Monica Concato, Emiliana Giacomello

**Affiliations:** 1 Laboratory of Muscle Biophysics, Department of Biomedical Sciences, University of Padova, 35131 Padova, Italy; 2 Department of Medicine, Surgery and Health Sciences, University of Trieste, 34149 Trieste, Italy; monica.concato@studenti.units.it

**Keywords:** resveratrol, skeletal muscle, clinical trials

## Abstract

Resveratrol is a natural polyphenol utilized in Chinese traditional medicine and thought to be one of the determinants of the “French Paradox”. More recently, some groups evidenced its properties as a calorie-restriction mimetic, suggesting that its action passes through the modulation of skeletal muscle metabolism. Accordingly, the number of studies reporting the beneficial effects of resveratrol on skeletal muscle form and function, in both experimental models and humans, is steadily increasing. Although studies on animal models confer to resveratrol a good potential to ameliorate skeletal muscle structure, function and performance, clinical trials still do not provide clear-cut information. Here, we first summarize the effects of resveratrol on the distinct components of the skeletal muscle, such as myofibers, the neuromuscular junction, tendons, connective sheaths and the capillary bed. Second, we review clinical trials focused on the analysis of skeletal muscle parameters. We suggest that the heterogeneity in the response to resveratrol in humans could depend on sample characteristics, treatment modalities and parameters analyzed; as well, this heterogeneity could possibly reside in the complexity of skeletal muscle physiology. A systematic programming of treatment protocols and analyses could be helpful to obtain consistent results in clinical trials involving resveratrol administration.

## 1. Introduction

**Figure 1:**
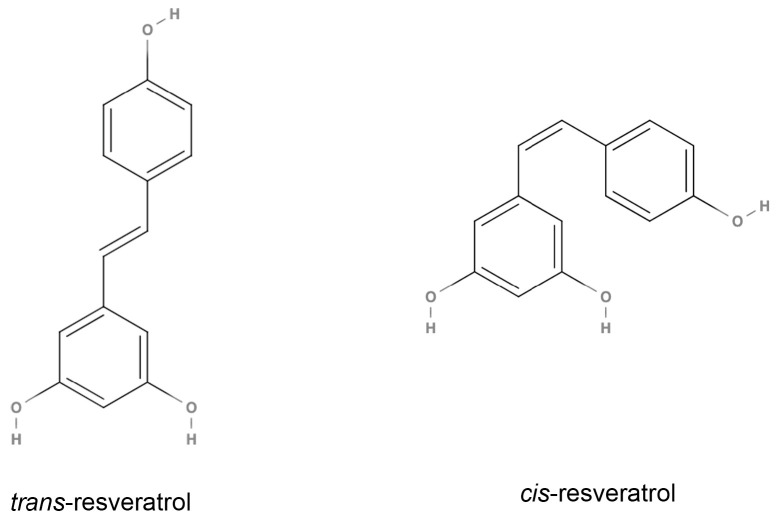
Structures of trans-resveratrol and cis-resveratrol.

Resveratrol (RES) is a natural polyphenol found in grapes, berries and peanuts (Figure 1). Utilized in Chinese traditional medicine, in more recent times, RES is thought to be one of the determinants of the so-called “French Paradox” [1]. This is an epidemiological observation that evidences a low mortality rate from cardiovascular diseases among people in France despite their diet rich in saturated fats [2]. At the beginning of this century, after the demonstration that RES can prolong the lifespan in several organisms by interacting with the Silent Mating Type Information Regulator 1 (SIRT-1) pathway [3,4,5], some groups evidenced its properties as an exercise and calorie-restriction mimetic [6]. After extensive research, it is now suggested that RES’s action is the result of the modulation of skeletal muscle cell metabolism and the improvement of mitochondrial quantity and quality, insulin sensitivity and motor function [7,8].

RES has been demonstrated to perform multiple functions at the level of the skeletal muscle, such as the modulation of cell metabolism, inhibition of protein catabolism and protection against cellular stress, thanks to its ability to interact with several signaling pathways [9,10]. Concisely, RES has been demonstrated to exert its metabolic effects mainly via the activation of adenosine monophosphate (AMP)-activated protein kinase (AMPK) and the consequent modulation of a signaling cascade involving SIRT-1, forkhead box O1 (FoxO1), nuclear factor erythroid-2-related factor 2 (Nrf2) and other effectors [10]. Successively, RES has been shown to regulate cyclooxygenases [11], to directly or indirectly scavenge reactive oxygen species (ROS) and nitrogen reactive species (RNS) [10] and to act as a phytoestrogen [12,13,14].

Although studies exploiting experimental models ascribe to RES a good potential to ameliorate skeletal muscle structure, function and performance [15,16], trials on humans still do not provide clear-cut information. Actually, in addition to the heterogeneity of protocols (sample number, dose and length of treatment, age, sex and health conditions), it should be considered that skeletal muscle function is achieved thanks to the orchestration of its distinct components, such as the neuromuscular junction (NMJ), muscle fibers, connective sheaths, tendons and vessels, which could be differently influenced by RES administration [17].

The present manuscript will provide an overview of the experimental evidence supporting RES action in the distinct components of the skeletal muscle and will summarize and discuss the recent literature on human trials that investigate the effects of RES on skeletal muscle form and function.

## 2. Resveratrol Can Affect Distinct Skeletal Muscle Components

Skeletal muscle contraction is initiated by the nervous system with the generation of a signal that travels through motor neurons to the NMJ, inducing the release of acetylcholine, which binds to receptors on the sarcolemma of the muscle fiber. This action starts a process, the excitation–contraction coupling, that leads to the filaments sliding and muscle contracting. Skeletal muscles are attached to bones and other structures via tendons, allowing movement and respiration. Although the main component of the skeletal muscle is represented by myofibers, an optimal physical performance depends on the integration of several histological and anatomical structures with different roles in skeletal muscle contraction. Attesting to the need for efficient coordination between myofibers and associated tissues to achieve optimal skeletal muscle function, studies on aging and muscle unloading demonstrate that the loss of muscle strength is considerably greater compared to the associated alteration to the muscle mass [18,19,20]. This divergence could be ascribable to modifications to the NMJ, connective sheets, tendons and blood vessels, which work together to guarantee optimal contraction performance [17]. Consequently, the efficient improvement of skeletal muscle function in different physio-pathological conditions requires a strategy capable of targeting multiple muscle structures [17]. In this context, RES, initially reported to modulate the form and function of the skeletal muscle cell [9], has been demonstrated to be a pleiotropic molecule able to interact with different muscle structures, as summarized below.

### 2.1. Resveratrol and Skeletal Muscle Fiber

RES has been reported to act on myofibers by modulating metabolism, catabolism and oxidative stress. As largely reported, RES interacts with the AMPK-SIRT-1 pathway to trigger several signaling pathways, which induces a general myofiber remodeling similar to that seen by exercise training and calorie restriction.

RES induces the expression of genes involved in mitochondrial biogenesis and oxidative phosphorylation through the peroxisome proliferator-activated receptor gamma coactivator-alpha (PGC-1-alpha) [7]. As a result, muscle fibers from treated animals have better oxidative profiles and/or present more oxidative type I fibers, which are resistant to fatigue [7,21]. In turn, mitochondrial activity and PGC-1-alpha modulation have been demonstrated to be strictly associated with better fatty acid oxidation and lipid metabolism [7].

RES has been shown to modulate glucose metabolism by improving glucose uptake in several experimental models. As a result, mice on a high-fat diet supplemented with RES have lower circulating levels of insulin and improved glucose tolerance compared to the control group [7,8], diabetic rats display a better glucose tolerance upon RES treatment [22,23] and, more interestingly, preliminary studies confer to RES potential as an antidiabetic molecule in humans [24].

Despite the presence in the literature of conflicting data, it is suggested that RES may prevent muscle wasting in different conditions. Actually, RES has been shown to inhibit protein degradation in several in vitro models [9] by interfering with nuclear factor kappa beta (NF-kB) activation and nuclear translocation, inhibiting the signaling pathway that contributes to muscle mass loss [25]. Accordingly, RES supplementation has been shown to attenuate age-dependent fiber area decrease [26]. Interestingly, the administration of RES in rats undergoing hindlimb suspension did not prevent fiber atrophy during the period of disuse, but it increased the cross-sectional area of type II fibers in response to reloading, most probably by reducing pro-apoptotic signals [27].

### 2.2. Resveratrol and the NMJ

The NMJ is the point of communication between the motor neuron and the skeletal muscle cell, and it is the site for the transmission of action potential to activate contraction. The integrity of the NMJ is perturbed in neuromuscular disorders and in skeletal muscle aging and disuse [20,28,29,30], with a consequent loss of its organization, fragmentation and degeneration, contributing to reduced muscle performance. Already in 2006, Lagouge and collaborators [7], based on the evidence that RES improved the motor coordination and traction force in mice fed on a high-fat diet, suggested that RES could modulate neuromuscular communication. Later, analogously to the reported evidence that calorie restriction and exercise can attenuate age-dependent NMJ modifications [31], RES was reported to slow aging of the NMJ by reducing its fragmentation and denervation [32].

Although the molecular pathways regulating the NMJ-specific domain targeted by RES remain quite unexplored (the research in PubMed with the words “resveratrol neuromuscular junction” issued only five results), several research articles exploring RES effects on nervous tissue reveal significant neuroprotective action. Actually, in various experimental models, RES administration has been shown to reduce nerve cell senescence [33] and to ameliorate oxidative stress [34], endoplasmic reticulum stress [35] and inflammation [36], thereby improving locomotor function. This evidence suggests that RES has the potential to target the presynaptic component of the NMJ. Further work is needed to understand if and how RES can target the postsynaptic domain of the skeletal muscle cell.

### 2.3. Resveratrol, Connective Sheaths and Tendons

Connective sheaths and tendons are formed by an insoluble scaffold of the extracellular matrix (ECM), rich in collagen and elastic fibers, proteoglycans, glycoproteins and laminins, which is synthesized by fibroblasts. Connective sheaths serve as structural support for muscle fibers; participate in lateral and longitudinal force transmission; host immune system cells, satellite cells, nerves and capillaries; and form a strict connection with bones to displace the different parts of the skeleton [37].

In general, ECM homeostasis is maintained by the fine regulation of the production of its components and the activation of degrading enzymes [18]. In the context of a muscle, the presence of continuous excitation–contraction cycles requires an appropriate remodeling of the ECM, which can be highly impacted by exercise, age and pathological conditions [37]. For example, in aging individuals, the ECM undergoes fibrotic changes that lead to an increase in skeletal muscle stiffness, strength loss and injury predisposition [19,38]. Moreover, attesting to the crucial role of connective sheaths, muscle contraction can be impaired in ECM-specific disorders but also in apparently unrelated pathologies that result in connective tissue impairment [39]. For instance, patients affected by Ehlers–Danlos syndrome often display neuromuscular involvement [40]. Although these subjects experience skeletal muscle symptoms such as pain, fatigue and cramps, they have normal skeletal muscles, suggesting that muscle symptoms depend on the associated connective tissue [41]. Interestingly, patients affected by chronic kidney disease present reduced muscle performance accompanied by fibrosis, capillary rarefaction and weakness, which can be partially reverted by dialysis [39,42].

The effects of RES on the ECM are quite conflicting, which may be due to the different biochemical properties of the ECM in different tissues and the experimental models tested. It has been suggested that RES may modulate both the deposition and the degradation of the ECM components. RES has been shown to increase collagen deposition, improving wound healing and neovascularization after laparotomy in rats [43] but also to reduce cardiac fibrosis in several experimental models via the diminution of ECM component deposition and the regulation of metalloproteinase (MMP) activity [44]. In the context of skeletal muscle, there are few observations. Gliemann and collaborators reported that RES inhibits the training-induced expression of the metallopeptidase inhibitor-1 (TIMP-1) and reduces the levels of thrombospondin-1 (TSP-1), and they interpreted these data as an inhibition of the proangiogenic response and capillarization [45]. Considering the metalloproteinase-inhibiting function of TIMP-1 and the role of TSP-1 in promoting fibrosis [46], it could be hypothesized that RES plays an antifibrotic role also in the skeletal muscle.

Interestingly, because RES is a pleiotropic molecule and ECM is a highly elaborate and plastic tissue structure, modifications to connective sheaths could also depend on indirect RES actions, such as the scavenging of ROS, inhibition of the production of advanced glycation end products (AGEs), improvement of the inflammatory response and regulation of hormones [47].

### 2.4. Resveratrol and Skeletal Muscle Vascularization

In the skeletal muscle, microcirculation is responsible for the delivery of oxygen, nutrition and hormone molecules to and from muscle fibers and for the removal of heat and waste products [48]. To these aims, within the skeletal muscle, fiber type, fiber size, oxidative capacity and capillarization are strictly regulated to adapt to physiological needs [48,49,50]. Attesting to its crucial role, reduced capillarization impacts oxygen supply to muscles, contributing to exercise intolerance. Accordingly, poor capillarization levels are observed in hypertensive conditions, concurrent with metabolic dysregulation [51] and associated with lower muscle performance in older adults [52].

Based on the idea of the capillary bed as a target to improve skeletal muscle health, there is a growing interest in RES’s potential to modulate capillarization, because studies in humans and animal models have provided promising results regarding both the skeletal muscle [53,54] and the cardiovascular system [55].

As suggested by Diaz and collaborators [56], the impact of RES on blood vessels is ascribable to a multilevel action, which starts from molecular regulation, passes through a biochemical response and converges to bring better blood supply to the tissue. RES has been shown to positively regulate vasculature through several mechanisms. It has been suggested that RES promotes angiogenesis via thioredoxin-1, heme oxygenase-1 and vascular endothelial growth factor (VEGF) [57] and regulates vasodilation by scavenging ROS and modulating nitric oxide synthesis. RES inhibits NFkB activation, leading to the reduction of inflammation markers and cytokines [53,58,59]. Ultimately, the improvement of capillarization in the skeletal muscle can also be credited with secondary effects, such as a decrease in fibrosis of the associated connective tissue [39,42] or an improvement in the cardiac hemodynamic properties, as shown in RES-treated diabetic rats [60].

## 3. Resveratrol and Skeletal Muscle in Clinical Trials

### 3.1. Search Strategy

We performed a systematic search from March 2023 to April 2023 following the Preferred Reporting Items for Systematic Reviews and Meta-Analyses (PRISMA) checklist guidelines. We searched the terms “resveratrol” and “skeletal muscle” in PubMed’s clinical trials section, which returned 21 results. We searched the terms “resveratrol”, “skeletal muscle” and “clinical trial” in Scopus, which returned 22 articles. We also searched the terms “resveratrol” and “skeletal muscle” in the Cochrane Library, and we obtained 53 results. We then selected only research articles that reported the analyses of skeletal muscle biopsies and/or physical performance. The cross-selection among libraries, together with a further analysis of the literature, returned 24 articles, which are reported in Table 1 [45,54,61,62,63,64,65,66,67,68,69,70,71,72,73,74,75,76,77,78,79,80,81,82]. Research articles from Gliemann et al. [45,78] and Olesen et al. [75], and research articles from Kjaer et al. [69] and Korsholm et al. [70], respectively, issued from a single clinical trial.

### 3.2. Effects of Resveratrol on Human Skeletal Muscle

Table 1 reports the 24 research articles issuing from 21 clinical trials that were selected from the literature. Interestingly, 14 articles reported positive or partially positive effects upon RES treatment, while 10 articles reported no beneficial action.

At the first evaluation, the analysis of the selected papers brings to light the wide heterogeneity of the clinical trials in the design, settings, participants, interventions and main outcomes.

The number of subjects ranged from less than 10 to around 20. The mean age of the subjects, although being quite homogeneous in the distinct trials, varied from around 20 years to a maximum of 75 years (see Table 1). The reported clinical trials prevalently enrolled male subjects, with one protocol involving only women [81] and seven protocols involving both female and male subjects [61,62,64,68,71,73,79]. Enrolled subjects were healthy active and healthy sedentary [45,61,65,68,73,75,76,77,78,79,81]; aged persons with functional limitations [62]; and subjects affected by obesity and/or metabolic syndrome [66,67,69,70,72,80,82], peripheral arterial disease (PAD) [71], type 2 diabetes mellitus (T2DM) [74,83] and chronic obstructive pulmonary disease (COPD) [64]. In most of the protocols, RES was supplemented orally, alone in pills, in combination with epigallocatechine-3 (EGCG) [72] or piperine [73], as a drink [61,79] and associated with training protocols [62,65,68,73,75,76,78]. The daily dose ranged from a minimum of 75 to a maximum of 2000–3000 mg/day [54,81] in a single treatment or for periods of 4 days to 6 months [61,77] (see Table 1).

Although we selected only research articles that reported the analyses of skeletal muscle biopsies and/or physical performance, there are significant differences also in the parameters and methodologies exploited to evaluate RES effectiveness. These include protein and gene expression of muscle proteins and the specific variations in known RES targets such as SIRT-1, PGC1-alpha, mitochondrial metabolism, glucose-related metabolism, inflammation markers, systemic parameters, histological properties and physical function.

There is also a good degree of variability concerning the components of skeletal muscle investigated and relative outcomes. In brief, most of the articles focused on the characteristics of muscle fibers, investigating morphology, mitochondrial function, lipid or glycogen content, metabolic properties and inflammatory conditions. Some works reported a RES-dependent modulation of the signaling pathways involving AMPK, SIRT-1 and PGC-1-alpha [62,65,74], while others did not [64,75,76].

Some articles explored the effectiveness of RES on the modification of vascularization. Pollack and collaborators showed that RES improves vascular function [54], while Gliemann and collaborators reported that RES does not improve angiogenic response and capillary growth [45]. Gliemann and collaborators quantified also some markers of connective tissue integrity, finding that RES inhibits the training-induced expression of TIMP-1 and reduces the levels of TSP-1 [45]. The histological properties of the NMJ were not evaluated in the reported clinical trials. The analysis of physical performance was monitored in six clinical trials, which reported either a positive or partially positive effect or no effect.

## 4. Discussion

Collectively, the reported clinical trials do not provide a clear-cut picture of the effectiveness of RES in improving skeletal muscle health. RES action on skeletal muscle can be modulated by several factors, which can be summarized as the age, sex, physical activity and health conditions of the subjects; the dose and length of the treatment; and, not last, the influence of the complexity of the histological and physiological components of this organ.

There are some elementary considerations that can influence the outcome of a clinical protocol, such as the probability of dealing with subjects with different genetic background [84,85], the possibility of sex-dependent action [14] and the evidence that although in the same chronological age window, individuals age differently. This intrinsic variance in the sample can play an important role in the effectiveness of RES supplementation, as demonstrated in aging rats, where RES effects on vascular functions and biomarkers are age- and gender-dependent [86]. Moreover, the population heterogeneity may imply disparities in the baseline skeletal muscle characteristics, which, in turn, can produce widely distributed outcomes. Therefore, contrary to experimental models that provide quite homogeneous samples, well-suited for both cross-sectional and longitudinal investigations, we should keep in mind that the intrinsic heterogeneity of human samples could lead to important variability in the results, requiring prudence in the evaluation of the potentiality of RES.

Similarly, health and nutritional conditions of the enrolled subjects could be crucial to note when observing an effect of RES treatment. It has been proposed that discrepancies in PGC-1alpha and UPC3 regulation during RES supplementation in aging mice could be ascribable to feeding conditions: only mice fed on a high-fat diet [7,87] experienced a significant change in these two pathways. This evidence, together with the hypothesis that RES exerts measurable beneficial effects only when cell metabolism properties are compromised, could explain the lack of RES effect in human trials involving young healthy adults [76,79], and on the other side, why obese, overweight and T2DM young subjects benefited from RES supplementation even with short periods of treatment [65].

The length and the time window of the treatment seems to be crucial for a positive effect. Again, experimental models are very useful in interpreting data. In our previous work, we showed that while long-term treatment with RES was able to ameliorate skeletal muscle tissue inflammation and improve capillarization in aging mice [21,53], short-term treatment only improved the inflammatory conditions with no effect on the capillaries [59]. In this context, some clinical trials reported partially positive effects, showing improvement in a few crucial parameters or resulting in a high variability in the response to RES treatment [69,71,72]. We cannot exclude the possibility that longer treatments could be more beneficial.

The daily doses chosen in the distinct trials, which ranged from a minimum of 75 to a maximum of 2000–3000 mg/day, might be a source of diverging outcomes. Although it is possible that the observations are the results of a J-shaped dose–response effect, evidence from several studies suggests a complex dose-dependent action, where RES can selectively interact with diverse signaling pathways at different dose levels, leading to different physiological outcomes [88,89]. Moreover, variations in RES bioavailability, which depends on several factors such as the administration modalities and variability of the examined subjects, can be a crucial variable in the response to RES. Interestingly, recent research demonstrating that molecular modifications to RES can increase its bioactivity opens up the possibility of investigating different RES-derived compounds also in humans [90,91]. However, to date, the heterogeneity of the clinical trials reported makes it difficult to advance hypotheses on the optimal dosage and the use of strategies that can promote absorption.

Among its multiple effects, RES has been reported to act as an exercise-mimetic molecule. Accordingly, six clinical trials (see Table 1) investigated the effects of RES administered together with a training protocol. Three clinical trials reported positive effects [62,68,73], while three reported either no beneficial effects or unfavorable effects of RES treatment [45,65,75,76,78]. These clinical trials are quite heterogeneous with regard to the subjects involved, the dose, the treatment length, the training protocol applied, the parameters measured and the outcome (Table 1). However, in this case, the quality of exercise seems to be a discriminating factor for RES effectiveness. RES supplementation together with walking and whole-body resistance training [62], resistance training combined with aerobic training [68] and low-intensity submaximal training [73] resulted in positive effects, which include fiber hypertrophy [68], attenuation of the proinflammatory state [62] and mitochondrial capacity [73]. On the contrary, high-intensity interval training (HIIT) did not show beneficial outcomes [45,65,75,76,78]. Work from Gliemann, Olesen and collaborators showed that HIIT, but not RES, improves the metabolic parameters, inflammatory state and capillary growth in the skeletal muscle and ameliorates cardiovascular health [45,75,78]. Analogously, Scribbans and collaborators showed that 4 weeks of RES supplementation together with HIIT does not improve the maximal oxygen uptake and anaerobic exercise capacity and abolishes the exercise-dependent increase in the skeletal muscle expression of PGC-1, SIRT1 and super oxide dismutase 2 (SOD2). These data suggest that RES may somewhat impair the adaptive response to HIIT in humans [76].

In the evaluation of RES effectiveness, it is worth taking into consideration that the heterogeneity of the results could also depend on the analyses performed to evaluate RES action. As reported in Table 1, protocols envisaged the analysis of physical activity, metabolism, inflammation or other markers. The choice of which parameters to analyze could be a key factor in establishing RES effectiveness in a clinical trial. In our hands, the treatment of aging mice with RES improved the resistance to fatigue of isolated muscles but did not induce a significant improvement in performance in a treadmill test, which displayed a high variability among the tested animals [21]. Most probably, the lack of improvement in the treadmill test is ascribable to other physiological factors and components that participate in physical activity. This could apply to data obtained by Mc Dermott and collaborators [71], who reported that 125 mg/day of RES induces a statistically significant but not a clinically meaningful improvement in a 6 min walk test in subjects affected by PAD. It would be interesting to know whether other parameters (metabolism, signaling pathways, etc.) changed in subjects enrolled in the protocol.

Of note, we observed heterogeneity also in the methods of quantification of chosen parameters. The mitochondrial activity has been measured with near infrared spectroscopy (NIRS), ex vivo or via mitochondrial markers. Protein expression has been investigated by capillary nano-immunoassays [62], Western blot analysis [74] and/or mRNA levels [65,75,76]. We cannot exclude the possibility that the different methodologies could be a further source of discrepancy in studies that demonstrate differing results in protein expression levels (i.e., mitochondrial markers). In this context, the work performed by Kjaer, Korsholm and collaborators is of interest, which indicated that metabolomic analyses of tissues could evidence differences not perceivable by analyzing standard biochemical parameters [69,70]. In addition, the investigation of humans limits the possibility of detecting time-dependent or transitory changes, which could explain some of the divergences observed. In fact, contrary to cross-sectional protocols in animal models that allow one to study the progression of modifications, human subjects cannot undergo multiple muscle biopsies, and cross-sectional studies are prone to high variability and have an important impact on the outcome.

In the evaluation of the global effectiveness of RES on skeletal muscle contraction and physical performance, it should be taken into account that muscle contraction and physical performance depend on the coordination of distinct components of muscles, which possibly have different levels of susceptibility to RES. It can be suggested that the complexity of skeletal muscle physiology, together with individual, health and age-related variability, could profoundly impact the outcome of physical performance tests, despite the positive effect of RES on specific signaling pathways and/or histological and metabolic parameters. It is of note that in the clinical trials reported here, some crucial components of the skeletal muscles, such as the connective sheaths and the NMJ, were not investigated or were poorly investigated, demonstrating the need for further studies.

As a final thought, we cannot exclude the possibility that RES could induce differential effects in metabolic organs and systemic parameters [92] that do not result in a dramatic change in skeletal muscle characteristics but that can anyway contribute to the health of this tissue.

## 5. Conclusions

The present review reveals a broad heterogeneity in the protocols of RES administration and the analysis of its effects, which induces caution in the evaluation of its effectiveness. In order to reach a consensus, it would be beneficial to apply systematic programming of human clinical trials and consequent evaluative work. Moreover, given the broad spectrum of RES targets that are in strict correlation with the skeletal muscles, such as the cardiovascular system, endocrine system and digestive apparatus, it would be advantageous to perform multitargeted trials involving collaborative groups with distinct interests, know-how and expertise. The association of systematic planning and analysis together with collaboration among groups with distinct expertise could contribute to obtaining reliable results.

## Figures and Tables

**Table 1:** List of the selected clinical trials reporting subjects’ number, sex and age (reported between parentheses as mean age ± standard deviation), health condition, treatment conditions, analyses performed and results. Abbreviations: f, female; m, male; RES, resveratrol; PL, placebo; EGCG, epigallocatechine-3; T2DM, type 2 diabetes mellitus; COPD, chronic obstructive pulmonary disease; and PAD, peripheral arterial disease.

	Groups Subjects No./Sex/Age	Health Condition	Dose	Treatment	Treatment Length	Parameters Measured	Effect
SCOTTO DI PALUMBO, 2022 [61]	10 f + 11 m RES (75.0 ± 3.4) 8 f + 8 m PL (74.8 ± 3.9)	Sedentary	150 mg/day	Nutrition supplement drink	6 months	Performance	Positive effect
HARPER, 2021 [62]	13 f + 7 m RES 500 mg (72 ± 5.1) 18 f + 2 m RES 1000 mg (70.3 ± 5.8) 14 f + 6 m PL (73.3 ± 7.8)	Functional limitations	500 mg, 1000 mg/day	Resveratrol + exercise	12 weeks	Metabolism, inflammation markers, performance	Positive effect
HUANG, 2021 [63]	18 m RES (21.09 ± 1.33) 18 m PL (21.09 ± 1.33)	T2DM	500 mg, 1000 mg/day	Trans-resveratrol extract	7 days	Muscle damage markers	Positive effect
BEIJERS, 2020 [64]	4 f + 7 m RES (67.8 ± 9.0) 5 f + 5 m PL (65.3 ± 9.1)	COPD	150 mg/day	resVidaTM	4 weeks	Metabolism, inflammation markers	No effect
HUANG, 2020 [65]	18 m RES (20.2 ± 0.4) 18 m PL (20.2 ± 0.4)	Healthy	480 mg/day	Resveratrol extract + exercise	4 days	Metabolism markers	No effect
VAN POLANEN, 2020 [66]	18 m RES (61 ± 8) 18 m PL (61 ± 8)	Obese Normoglycemic	150 mg/day	Trans-resveratrol	30 days	Metabolism, histological markers	Positive effect
DE LIGT, 2018 [67]	13 m RES (66) 13 m PL (66)	Overweight (T2DM first-degree relatives)	150 mg/day	Trans-resveratrol	30–34 days	Metabolism markers, respiration	Partially positive effect
ALWAY, 2017 [68]	9 f + 6 m RES (67.9 ± 1.1) 9 f + 6 m PL (67.9 ± 1.1)	Healthy	500 mg/day	Resveratrol + exercise	12 weeks	Metabolism, histological markers, performance	Positive effect
KJAER, 2017 [69]	21 m RES 150 mg (49.1 ± 1.46) 21 m RES 1000 mg (51.9 ± 1.28) 24 m PL (47.8 ± 1.3)	Metabolic syndrome	150 mg 1000 mg/day	Resveratrol	16 weeks	Metabolism markers	No effect
KORSHOLM, 2017 [70]	21 m RES (47.8 ± 1.3) 24 m PL (47.8 ± 1.3)	Metabolic syndrome	1000 mg/day	resVidaTM	4 months	Metabolomic analyses	Positive effect
MCDERMOTT, 2017 [71]	8 f + 13 m RES 125 mg (73.6 ± 6.6) 6 f + 17 m RES 500 mg (75.6 ± 7.3) 21 f + 45 m PL (74.4 ± 6.1)	PAD	125, 500 mg/day	Resveratrol	6 months	Performance	Partially positive effect
POLLACK, 2017 [54]	19 f (67 ± 7) 11 m (67 ± 7)	Glucose intolerant	2000–3000 mg/day	Resveratrol	6 weeks	Metabolism markers	Positive effect
MOST, 2016 [72]	18 EGCG + RES (36.1 ± 2.2) 20 PL (38.7 ± 2.2)	Overweight Obese	80 mg/day	Trans-resveratrol extract + epigallocatechine-3	12 weeks	Metabolism markers, respiration	Positive effects
POLLEY, 2016 [73]	4 f (19.7 ± 0.6) + 4 m (21.07 ± 2.4) RES 3 f (19.0 ± 0.8) + 5 m (20.0 ± 0.8) PL	Healthy	500 mg/day	Trans-resveratrol + 10mg/day piperine + exercise	4 weeks	Metabolism markers, respiration	Positive effects
GLIEMANN, 2014 [45]	9 m RES (60–72) 14 m RES + exercise (60–72) 7 m PL (60–72) 13 m PL + exercise (60–72)	Healthy	250 mg/day	Trans-resveratrol + exercise	8 weeks	Angiogenic markers	No effect
GOH, 2014 [74]	5 m RES (55.8 ± 7.3) 5 m PL (56.8 ± 5.3)	T2DM	500 mg/day	Trans-resveratrol	12 weeks	Metabolisms markers, performance	Partially positive effect
OLESEN, 2014 [75]	9 m RES (60–72) 14 m RES + exercise (60–72) 7 m PL (60–72) 13 m PL + exercise (60–72)	Healthy	250 mg/day	Trans-resveratrol + exercise	8 weeks	Metabolism, inflammation markers	No effect
SCRIBBANS, 2014 [76]	8 m RES (21 ± 1) 8 m PL (22 ± 1)	Healthy	150 mg/day	resVidaTM + exercise	4 weeks	Metabolism, histological markers, performance	Partially positive effect
WILLIAMS, 2014 [77]	8 m RES (23.8 ± 2.4) 8 m PL (23.4 ± 6.1)	Sedentary	300 mg	resVidaTM	Single dose	Metabolism markers, respiration	No effect
GLIEMANN, 2013 [78]	9 m RES (60–72) 14 m RES + exercise (60–72) 7 m PL (60–72) 13 m PL + exercise (60–72)	Healthy	250 mg/day	Trans-resveratrol + exercise	8 weeks	Metabolism, inflammation markers	No effect
O’CONNOR, 2013 [79]	9 f + 11 m RES (19.9 ± 2.2) 12 f + 10 m PL (19.8 ± 1.7)	Healthy		Grape powder drink	45 days	Muscle performance, injury	No effect
POULSEN, 2013 [80]	12 m RES (44.7 ± 3.5) 12 m PL (31.1 ± 3.9)	Obese	1500 mg/day	Trans-resveratrol	4 weeks	Metabolism markers	No effect
YOSHINO, 2012 [81]	15 f RES (58.2.5 ± 4.0) 14 f PL (59.8.5 ± 4.3)	Healthy, postmenopausal	75 mg/day	Resveratrol	12 weeks	Metabolism markers	No effect
TIMMERS, 2011 [82]	11 m RES (52.5 ± 2.1) 11 m PL (52.5 ± 2.1)	Obese	150 mg/day	resVidaTM	30 days	Metabolism markers	Positive effect

## Data Availability

Not applicable.

## References

[1] Cordova A.C., Jackson L.S.M., Berke-Schlessel D.W., Sumpio B.E. (2005). The Cardiovascular Protective Effect of Red Wine. J. Am. Coll. Surg..

[2] Renaud S., de Lorgeril M. (1992). Wine, Alcohol, Platelets, and the French Paradox for Coronary Heart Disease. Lancet.

[3] Valenzano D.R., Terzibasi E., Genade T., Cattaneo A., Domenici L., Cellerino A. (2006). Resveratrol Prolongs Lifespan and Retards the Onset of Age-Related Markers in a Short-Lived Vertebrate. Curr. Biol..

[4] Howitz K.T., Bitterman K.J., Cohen H.Y., Lamming D.W., Lavu S., Wood J.G., Zipkin R.E., Chung P., Kisielewski A., Zhang L.-L. (2003). Small Molecule Activators of Sirtuins Extend Saccharomyces Cerevisiae Lifespan. Nature.

[5] Wood J.G., Rogina B., Lavu S., Howitz K., Helfand S.L., Tatar M., Sinclair D. (2004). Sirtuin Activators Mimic Caloric Restriction and Delay Ageing in Metazoans. Nature.

[6] Chung J.H., Manganiello V., Dyck J.R.B. (2012). Resveratrol as a Calorie Restriction Mimetic: Therapeutic Implications. Trends Cell Biol..

[7] Lagouge M., Argmann C., Gerhart-Hines Z., Meziane H., Lerin C., Daussin F., Messadeq N., Milne J., Lambert P., Elliott P. (2006). Resveratrol Improves Mitochondrial Function and Protects against Metabolic Disease by Activating SIRT1 and PGC-1α. Cell.

[8] Baur J.A., Pearson K.J., Price N.L., Jamieson H.A., Lerin C., Kalra A., Prabhu V.V., Allard J.S., Lopez-Lluch G., Lewis K. (2006). Resveratrol Improves Health and Survival of Mice on a High-Calorie Diet. Nature.

[9] Dirks Naylor A.J. (2009). Cellular Effects of Resveratrol in Skeletal Muscle. Life Sci..

[10] Meng X., Zhou J., Zhao C.-N., Gan R.-Y., Li H.-B. (2020). Health Benefits and Molecular Mechanisms of Resveratrol: A Narrative Review. Foods.

[11] Jang M., Cai L., Udeani G.O., Slowing K.V., Thomas C.F., Beecher C.W.W., Fong H.H.S., Farnsworth N.R., Kinghorn A.D., Mehta R.G. (1997). Cancer Chemopreventive Activity of Resveratrol, a Natural Product Derived from Grapes. Science.

[12] Kulkarni S.S., Cantó C. (2015). The Molecular Targets of Resveratrol. Biochim. Biophys. Acta (BBA) Mol. Basis Dis..

[13] Pezzuto J.M. (2011). The Phenomenon of Resveratrol: Redefining the Virtues of Promiscuity. Ann. N. Y. Acad. Sci..

[14] Gehm B.D., McAndrews J.M., Chien P.-Y., Jameson J.L. (1997). Resveratrol, a Polyphenolic Compound Found in Grapes and Wine, Is an Agonist for the Estrogen Receptor. Proc. Natl. Acad. Sci. USA.

[15] Giacomello E., Toniolo L. (2021). The Potential of Calorie Restriction and Calorie Restriction Mimetics in Delaying Aging: Focus on Experimental Models. Nutrients.

[16] Toniolo L., Giacomello E. (2020). Resveratrol, Aging, and Fatigue. Aging: Oxidative Stress and Dietary Antioxidants.

[17] Tieland M., Trouwborst I., Clark B.C. (2018). Skeletal Muscle Performance and Ageing. J. Cachexia Sarcopenia Muscle.

[18] Kumar L., Bisen M., Khan A., Kumar P., Patel S.K.S. (2022). Role of Matrix Metalloproteinases in Musculoskeletal Diseases. Biomedicines.

[19] Sinha U., Malis V., Chen J.-S., Csapo R., Kinugasa R., Narici M.V., Sinha S. (2020). Role of the Extracellular Matrix in Loss of Muscle Force with Age and Unloading Using Magnetic Resonance Imaging, Biochemical Analysis, and Computational Models. Front. Physiol..

[20] Sirago G., Pellegrino M.A., Bottinelli R., Franchi M.V., Narici M.V. (2023). Loss of Neuromuscular Junction Integrity and Muscle Atrophy in Skeletal Muscle Disuse. Ageing Res. Rev..

[21] Toniolo L., Fusco P., Formoso L., Mazzi A., Canato M., Reggiani C., Giacomello E. (2018). Resveratrol Treatment Reduces the Appearance of Tubular Aggregates and Improves the Resistance to Fatigue in Aging Mice Skeletal Muscles. Exp. Gerontol..

[22] Chi T.-C., Chen W.-P., Chi T.-L., Kuo T.-F., Lee S.-S., Cheng J.-T., Su M.-J. (2007). Phosphatidylinositol-3-Kinase Is Involved in the Antihyperglycemic Effect Induced by Resveratrol in Streptozotocin-Induced Diabetic Rats. Life Sci..

[23] Su H.-C., Hung L.-M., Chen J.-K. (2006). Resveratrol, a Red Wine Antioxidant, Possesses an Insulin-like Effect in Streptozotocin-Induced Diabetic Rats. Am. J. Physiol.-Endocrinol. Metab..

[24] Szkudelski T., Szkudelska K. (2015). Resveratrol and Diabetes: From Animal to Human Studies. Biochim. Biophys. Acta (BBA) Mol. Basis Dis..

[25] Li H., Malhotra S., Kumar A. (2008). Nuclear Factor-Kappa B Signaling in Skeletal Muscle Atrophy. J. Mol. Med..

[26] Hosoda R., Nakashima R., Yano M., Iwahara N., Asakura S., Nojima I., Saga Y., Kunimoto R., Horio Y., Kuno A. (2023). Resveratrol, a SIRT1 Activator, Attenuates Aging-Associated Alterations in Skeletal Muscle and Heart in Mice. J. Pharmacol. Sci..

[27] Bennett B.T., Mohamed J.S., Alway S.E. (2013). Effects of Resveratrol on the Recovery of Muscle Mass Following Disuse in the Plantaris Muscle of Aged Rats. PLoS ONE.

[28] Monti E., Reggiani C., Franchi M.V., Toniolo L., Sandri M., Armani A., Zampieri S., Giacomello E., Sarto F., Sirago G. (2021). Neuromuscular Junction Instability and Altered Intracellular Calcium Handling as Early Determinants of Force Loss during Unloading in Humans. J. Physiol..

[29] Sirago G., Candia J., Franchi M.V., Sarto F., Monti E., Toniolo L., Reggiani C., Giacomello E., Zampieri S., Hartnell L.M. (2023). Upregulation of Sarcolemmal Hemichannels and Inflammatory Transcripts with Neuromuscular Junction Instability during Lower Limb Unloading in Humans. Biology.

[30] Gonzalez-Freire M., de Cabo R., Studenski S.A., Ferrucci L. (2014). The Neuromuscular Junction: Aging at the Crossroad between Nerves and Muscle. Front. Aging Neurosci..

[31] Valdez G., Tapia J.C., Kang H., Clemenson G.D., Gage F.H., Lichtman J.W., Sanes J.R. (2010). Attenuation of Age-Related Changes in Mouse Neuromuscular Synapses by Caloric Restriction and Exercise. Proc. Natl. Acad. Sci. USA.

[32] Stockinger J., Maxwell N., Shapiro D., deCabo R., Valdez G. (2018). Caloric Restriction Mimetics Slow Aging of Neuromuscular Synapses and Muscle Fibers. J. Gerontol. A Biol. Sci. Med. Sci..

[33] Liu J., Jiao K., Zhou Q., Yang J., Yang K., Hu C., Zhou M., Li Z. (2021). Resveratrol Alleviates 27-Hydroxycholesterol-Induced Senescence in Nerve Cells and Affects Zebrafish Locomotor Behavior via Activation of SIRT1-Mediated STAT3 Signaling. Oxid. Med. Cell Longev..

[34] Adedara A.O., Babalola A.D., Stephano F., Awogbindin I.O., Olopade J.O., Rocha J.B.T., Whitworth A.J., Abolaji A.O. (2022). An Assessment of the Rescue Action of Resveratrol in Parkin Loss of Function-Induced Oxidative Stress in Drosophila Melanogaster. Sci. Rep..

[35] Luo Y., Zhao Y., Lai J., Wei L., Zhou G., Yu Y., Liu J. (2023). Resveratrol Suppresses Bupivacaine-Induced Spinal Neurotoxicity in Rats by Inhibiting Endoplasmic Reticulum Stress via SIRT1 Modulation. Biomed. Res. Int..

[36] Zhao H., Mei X., Yang D., Tu G. (2021). Resveratrol Inhibits Inflammation after Spinal Cord Injury via SIRT-1/NF-ΚB Signaling Pathway. Neurosci. Lett..

[37] Csapo R., Gumpenberger M., Wessner B. (2020). Skeletal Muscle Extracellular Matrix–What Do We Know About Its Composition, Regulation, and Physiological Roles? A Narrative Review. Front. Physiol..

[38] Lieber R.L., Ward S.R. (2013). Cellular Mechanisms of Tissue Fibrosis. 4. Structural and Functional Consequences of Skeletal Muscle Fibrosis. Am. J. Physiol.-Cell Physiol..

[39] Brightwell C.R., Kulkarni A.S., Paredes W., Zhang K., Perkins J.B., Gatlin K.J., Custodio M., Farooq H., Zaidi B., Pai R. (2021). Muscle Fibrosis and Maladaptation Occur Progressively in CKD and Are Rescued by Dialysis. JCI Insight.

[40] Voermans N.C., van Alfen N., Pillen S., Lammens M., Schalkwijk J., Zwarts M.J., van Rooij I.A., Hamel B.C.J., van Engelen B.G. (2009). Neuromuscular Involvement in Various Types of Ehlers–Danlos Syndrome. Ann. Neurol..

[41] Nygaard R.H., Jensen J.K., Voermans N.C., Heinemeier K.M., Schjerling P., Holm L., Agergaard J., Mackey A.L., Andersen J.L., Remvig L. (2017). Skeletal Muscle Morphology, Protein Synthesis, and Gene Expression in Ehlers-Danlos Syndrome. J. Appl. Physiol..

[42] Abramowitz M.K., Paredes W., Zhang K., Brightwell C.R., Newsom J.N., Kwon H.-J., Custodio M., Buttar R.S., Farooq H., Zaidi B. (2018). Skeletal Muscle Fibrosis Is Associated with Decreased Muscle Inflammation and Weakness in Patients with Chronic Kidney Disease. Am. J. Physiol.-Ren. Physiol..

[43] Yaman I., Derici H., Kara C., Kamer E., Diniz G., Ortac R., Sayin O. (2013). Effects of Resveratrol on Incisional Wound Healing in Rats. Surg. Today.

[44] Yu D., Tang Z., Li B., Yu J., Li W., Liu Z., Tian C. (2021). Resveratrol against Cardiac Fibrosis: Research Progress in Experimental Animal Models. Molecules.

[45] Gliemann L., Olesen J., Biensø R.S., Schmidt J.F., Akerstrom T., Nyberg M., Lindqvist A., Bangsbo J., Hellsten Y. (2014). Resveratrol Modulates the Angiogenic Response to Exercise Training in Skeletal Muscles of Aged Men. Am. J. Physiol.-Heart Circ. Physiol..

[46] Xu L., Zhang Y., Chen J., Xu Y. (2020). Thrombospondin-1: A Key Protein That Induces Fibrosis in Diabetic Complications. J. Diabetes Res..

[47] Agarwal R., Agarwal P. (2017). Targeting Extracellular Matrix Remodeling in Disease: Could Resveratrol Be a Potential Candidate?. Exp. Biol. Med..

[48] Hendrickse P., Degens H. (2019). The Role of the Microcirculation in Muscle Function and Plasticity. J. Muscle Res. Cell Motil..

[49] Giacomello E., Crea E., Torelli L., Bergamo A., Reggiani C., Sava G., Toniolo L. (2020). Age Dependent Modification of the Metabolic Profile of the Tibialis Anterior Muscle Fibers in C57BL/6J Mice. Int. J. Mol. Sci..

[50] Barnouin Y., McPhee J.S., Butler-Browne G., Bosutti A., De Vito G., Jones D.A., Narici M., Behin A., Hogrel J., Degens H. (2017). Coupling between Skeletal Muscle Fiber Size and Capillarization Is Maintained during Healthy Aging. J. Cachexia Sarcopenia Muscle.

[51] Gueugneau M., Coudy-Gandilhon C., Meunier B., Combaret L., Taillandier D., Polge C., Attaix D., Roche F., Féasson L., Barthélémy J.-C. (2016). Lower Skeletal Muscle Capillarization in Hypertensive Elderly Men. Exp. Gerontol..

[52] Prior S.J., Ryan A.S., Blumenthal J.B., Watson J.M., Katzel L.I., Goldberg A.P. (2016). Sarcopenia Is Associated with Lower Skeletal Muscle Capillarization and Exercise Capacity in Older Adults. J. Gerontol. A Biol. Sci. Med. Sci..

[53] Toniolo L., Formoso L., Torelli L., Crea E., Bergamo A., Sava G., Giacomello E. (2021). Long-Term Resveratrol Treatment Improves the Capillarization in the Skeletal Muscles of Ageing C57BL/6J Mice. Int. J. Food Sci. Nutr..

[54] Pollack R.M., Barzilai N., Anghel V., Kulkarni A.S., Golden A., O’Broin P., Sinclair D.A., Bonkowski M.S., Coleville A.J., Powell D. (2017). Resveratrol Improves Vascular Function and Mitochondrial Number but Not Glucose Metabolism in Older Adults. J. Gerontol. A Biol. Sci. Med. Sci..

[55] Dolinsky V.W., Dyck J.R.B. (2014). Experimental Studies of the Molecular Pathways Regulated by Exercise and Resveratrol in Heart, Skeletal Muscle and the Vasculature. Molecules.

[56] Diaz M., Degens H., Vanhees L., Austin C., Azzawi M. (2016). The Effects of Resveratrol on Aging Vessels. Exp. Gerontol..

[57] Kaga S., Zhan L., Matsumoto M., Maulik N. (2005). Resveratrol Enhances Neovascularization in the Infarcted Rat Myocardium through the Induction of Thioredoxin-1, Heme Oxygenase-1 and Vascular Endothelial Growth Factor. J. Mol. Cell. Cardiol..

[58] Pearson K.J., Baur J.A., Lewis K.N., Peshkin L., Price N.L., Labinskyy N., Swindell W.R., Kamara D., Minor R.K., Perez E. (2008). Resveratrol Delays Age-Related Deterioration and Mimics Transcriptional Aspects of Dietary Restriction without Extending Life Span. Cell Metab..

[59] Sirago G., Toniolo L., Crea E., Giacomello E. (2022). A Short-Term Treatment with Resveratrol Improves the Inflammatory Conditions of Middle-Aged Mice Skeletal Muscles. Int. J. Food Sci. Nutr..

[60] Bresciani L., Calani L., Bocchi L., Delucchi F., Savi M., Ray S., Brighenti F., Stilli D., Del Rio D. (2014). Bioaccumulation of Resveratrol Metabolites in Myocardial Tissue Is Dose-Time Dependent and Related to Cardiac Hemodynamics in Diabetic Rats. Nutr. Metab. Cardiovasc. Dis..

[61] Scotto di Palumbo A., McSwiney F.T., Hone M., McMorrow A.M., Lynch G., De Vito G., Egan B. (2022). Effects of a Long Chain N-3 Polyunsaturated Fatty Acid-Rich Multi-Ingredient Nutrition Supplement on Body Composition and Physical Function in Older Adults with Low Skeletal Muscle Mass. J. Diet. Suppl..

[62] Harper S.A., Bassler J.R., Peramsetty S., Yang Y., Roberts L.M., Drummer D., Mankowski R.T., Leeuwenburgh C., Ricart K., Patel R.P. (2021). Resveratrol and Exercise Combined to Treat Functional Limitations in Late Life: A Pilot Randomized Controlled Trial. Exp. Gerontol..

[63] Huang B., Li X., Zhu X. (2021). The Role of GM130 in Nervous System Diseases. Front. Neurol..

[64] Beijers R.J.H.C.G., Gosker H.R., Sanders K.J.C., de Theije C., Kelders M., Clarke G., Cryan J.F., van den Borst B., Schols A.M.W.J. (2020). Resveratrol and Metabolic Health in COPD: A Proof-of-Concept Randomized Controlled Trial. Clin. Nutr..

[65] Huang C.-C., Liu C.-C., Tsao J.-P., Hsu C.-L., Cheng I.-S. (2020). Effects of Oral Resveratrol Supplementation on Glycogen Replenishment and Mitochondria Biogenesis in Exercised Human Skeletal Muscle. Nutrients.

[66] Van Polanen N., Zacharewicz E., de Ligt M., Timmers S., Moonen-Kornips E., Schaart G., Hoeks J., Schrauwen P., Hesselink M.K.C. (2021). Resveratrol-induced Remodelling of Myocellular Lipid Stores: A Study in Metabolically Compromised Humans. Physiol. Rep..

[67] De Ligt M., Bruls Y.M.H., Hansen J., Habets M.-F., Havekes B., Nascimento E.B.M., Moonen-Kornips E., Schaart G., Schrauwen-Hinderling V.B., van Marken Lichtenbelt W. (2018). Resveratrol Improves Ex Vivo Mitochondrial Function but Does Not Affect Insulin Sensitivity or Brown Adipose Tissue in First Degree Relatives of Patients with Type 2 Diabetes. Mol. Metab..

[68] Alway S.E., McCrory J.L., Kearcher K., Vickers A., Frear B., Gilleland D.L., Bonner D.E., Thomas J.M., Donley D.A., Lively M.W. (2017). Resveratrol Enhances Exercise-Induced Cellular and Functional Adaptations of Skeletal Muscle in Older Men and Women. J. Gerontol. A Biol. Sci. Med. Sci..

[69] Kjær T.N., Ornstrup M.J., Poulsen M.M., Stødkilde-Jørgensen H., Jessen N., Jørgensen J.O.L., Richelsen B., Pedersen S.B. (2017). No Beneficial Effects of Resveratrol on the Metabolic Syndrome: A Randomized Placebo-Controlled Clinical Trial. J. Clin. Endocrinol. Metab..

[70] Korsholm A.S., Kjær T.N., Ornstrup M.J., Pedersen S.B. (2017). Comprehensive Metabolomic Analysis in Blood, Urine, Fat, and Muscle in Men with Metabolic Syndrome: A Randomized, Placebo-Controlled Clinical Trial on the Effects of Resveratrol after Four Months’ Treatment. Int. J. Mol. Sci..

[71] McDermott M.M., Leeuwenburgh C., Guralnik J.M., Tian L., Sufit R., Zhao L., Criqui M.H., Kibbe M.R., Stein J.H., Lloyd-Jones D. (2017). Effect of Resveratrol on Walking Performance in Older People With Peripheral Artery Disease. JAMA Cardiol..

[72] Most J., Timmers S., Warnke I., Jocken J.W., van Boekschoten M., de Groot P., Bendik I., Schrauwen P., Goossens G.H., Blaak E.E. (2016). Combined Epigallocatechin-3-Gallate and Resveratrol Supplementation for 12 Wk Increases Mitochondrial Capacity and Fat Oxidation, but Not Insulin Sensitivity, in Obese Humans: A Randomized Controlled Trial1,2. Am. J. Clin. Nutr..

[73] Polley K.R., Jenkins N., O’Connor P., McCully K. (2016). Influence of Exercise Training with Resveratrol Supplementation on Skeletal Muscle Mitochondrial Capacity. Appl. Physiol. Nutr. Metab..

[74] Goh K.P., Lee H.Y., Lau D.P., Supaat W., Chan Y.H., Koh A.F.Y. (2014). Effects of Resveratrol in Patients with Type 2 Diabetes Mellitus on Skeletal Muscle SIRT1 Expression and Energy Expenditure. Int. J. Sport. Nutr. Exerc. Metab..

[75] Olesen J., Gliemann L., Biensø R., Schmidt J., Hellsten Y., Pilegaard H. (2014). Exercise Training, but Not Resveratrol, Improves Metabolic and Inflammatory Status in Skeletal Muscle of Aged Men. J. Physiol..

[76] Scribbans T.D., Ma J.K., Edgett B.A., Vorobej K.A., Mitchell A.S., Zelt J.G.E., Simpson C.A., Quadrilatero J., Gurd B.J. (2014). Resveratrol Supplementation Does Not Augment Performance Adaptations or Fibre-Type–Specific Responses to High-Intensity Interval Training in Humans. Appl. Physiol. Nutr. Metab..

[77] Williams C.B., Hughes M.C., Edgett B.A., Scribbans T.D., Simpson C.A., Perry C.G.R., Gurd B.J. (2014). An Examination of Resveratrol’s Mechanisms of Action in Human Tissue: Impact of a Single Dose In Vivo and Dose Responses in Skeletal Muscle Ex Vivo. PLoS ONE.

[78] Gliemann L., Schmidt J.F., Olesen J., Biensø R.S., Peronard S.L., Grandjean S.U., Mortensen S.P., Nyberg M., Bangsbo J., Pilegaard H. (2013). Resveratrol Blunts the Positive Effects of Exercise Training on Cardiovascular Health in Aged Men. J. Physiol..

[79] O’Connor P.J., Caravalho A.L., Freese E.C., Cureton K.J. (2013). Grape Consumption’s Effects on Fitness, Muscle Injury, Mood, and Perceived Health. Int. J. Sport. Nutr. Exerc. Metab..

[80] Poulsen M.M., Vestergaard P.F., Clasen B.F., Radko Y., Christensen L.P., Stødkilde-Jørgensen H., Møller N., Jessen N., Pedersen S.B., Jørgensen J.O.L. (2013). High-Dose Resveratrol Supplementation in Obese Men: An Investigator-Initiated, Randomized, Placebo-Controlled Clinical Trial of Substrate Metabolism, Insulin Sensitivity, and Body Composition. Diabetes.

[81] Yoshino J., Conte C., Fontana L., Mittendorfer B., Imai S., Schechtman K.B., Gu C., Kunz I., Fanelli F.R., Patterson B.W. (2012). Resveratrol Supplementation Does Not Improve Metabolic Function in Non-Obese Women with Normal Glucose Tolerance. Cell Metab..

[82] Timmers S., Konings E., Bilet L., Houtkooper R.H., van de Weijer T., Goossens G.H., Hoeks J., van der Krieken S., Ryu D., Kersten S. (2011). Calorie Restriction-like Effects of 30 Days of Resveratrol (ResVida^TM^) Supplementation on Energy Metabolism and Metabolic Profile in Obese Humans. Cell Metab..

[83] Huang C.-C., Lee M.-C., Ho C.-S., Hsu Y.-J., Ho C.-C., Kan N.-W. (2021). Protective and Recovery Effects of Resveratrol Supplementation on Exercise Performance and Muscle Damage Following Acute Plyometric Exercise. Nutrients.

[84] Murgia M., Brocca L., Monti E., Franchi M.V., Zwiebel M., Steigerwald S., Giacomello E., Sartori R., Zampieri S., Capovilla G. (2022). Plasma Proteome Profiling of Healthy Subjects Undergoing Bed Rest Reveals Unloading-dependent Changes Linked to Muscle Atrophy. J. Cachexia Sarcopenia Muscle.

[85] Sirago G., Picca A., Giacomello E., Marzetti E., Toniolo L. (2022). The Contribution of Genetics to Muscle Disuse, Retraining, and Aging. Genes.

[86] Han S., Bal N.B., Sadi G., Usanmaz S.E., Uludag M.O., Demirel-Yilmaz E. (2018). The Effects of Resveratrol and Exercise on Age and Gender-Dependent Alterations of Vascular Functions and Biomarkers. Exp. Gerontol..

[87] Barger J.L., Kayo T., Vann J.M., Arias E.B., Wang J., Hacker T.A., Wang Y., Raederstorff D., Morrow J.D., Leeuwenburgh C. (2008). A Low Dose of Dietary Resveratrol Partially Mimics Caloric Restriction and Retards Aging Parameters in Mice. PLoS ONE.

[88] Bosutti A., Degens H. (2015). The Impact of Resveratrol and Hydrogen Peroxide on Muscle Cell Plasticity Shows a Dose-Dependent Interaction. Sci. Rep..

[89] Cho S.-J., Jung U.J., Choi M.-S. (2012). Differential Effects of Low-Dose Resveratrol on Adiposity and Hepatic Steatosis in Diet-Induced Obese Mice. Br. J. Nutr..

[90] Nawaz W., Zhou Z., Deng S., Ma X., Ma X., Li C., Shu X. (2017). Therapeutic Versatility of Resveratrol Derivatives. Nutrients.

[91] Biasutto L., Mattarei A., Azzolini M., La Spina M., Sassi N., Romio M., Paradisi C., Zoratti M. (2017). Resveratrol Derivatives as a Pharmacological Tool. Ann. N. Y. Acad. Sci..

[92] Svensson K., Schnyder S., Albert V., Cardel B., Quagliata L., Terracciano L.M., Handschin C. (2015). Resveratrol and SRT1720 Elicit Differential Effects in Metabolic Organs and Modulate Systemic Parameters Independently of Skeletal Muscle Peroxisome Proliferator-Activated Receptor γ Co-Activator 1α (PGC-1α). J. Biol. Chem..

